# A Self-Emulsified Adjuvant System Containing the Immune Potentiator Alpha Tocopherol Induces Higher Neutralizing Antibody Responses than a Squalene-Only Emulsion When Evaluated with a Recombinant Cytomegalovirus (CMV) Pentamer Antigen in Mice

**DOI:** 10.3390/pharmaceutics15010238

**Published:** 2023-01-10

**Authors:** Rushit N. Lodaya, Amey P. Kanitkar, Asma Ashraf, Douty Bamba, Mansoor M. Amiji, Derek T. O’Hagan

**Affiliations:** 1 Department of Pharmaceutical Sciences, School of Pharmacy, Northeastern University, Boston, MA 02115, USA; 2 GSK, Rockville Centre for Vaccines Research, Rockville, MD 20850, USA

**Keywords:** adjuvants, alpha-tocopherol, nanoemulsions, vaccine delivery, self-emulsification

## Abstract

The development of new vaccine adjuvants represents a key approach to improvingi the immune responses to recombinant vaccine antigens. Emulsion adjuvants, such as AS03 and MF59, in combination with influenza vaccines, have allowed antigen dose sparing, greater breadth of responses and fewer immunizations. It has been demonstrated previously that emulsion adjuvants can be prepared using a simple, low-shear process of self-emulsification (SE). The role of alpha tocopherol as an immune potentiator in emulsion adjuvants is clear from the success of AS03 in pandemic responses, both to influenza and COVID-19. Although it was a significant formulation challenge to include alpha tocopherol in an emulsion prepared by a low-shear process, the resultant self-emulsifying adjuvant system (SE-AS) showed a comparable effect to the established AS03 when used with a quadrivalent influenza vaccine (QIV). In this paper, we first optimized the SE-AS with alpha tocopherol to create SE-AS44, which allowed the emulsion to be sterile-filtered. Then, we compared the in vitro cell activation cytokine profile of SE-AS44 with the self-emulsifying adjuvant 160 (SEA160), a squalene-only adjuvant. In addition, we evaluated SE-AS44 and SEA160 competitively, in combination with a recombinant cytomegalovirus (CMV) pentamer antigen mouse.

## 1. Introduction

Emulsion adjuvants are well-established as safe and effective when combined with a variety of antigens, particularly influenza vaccines for both seasonal and pandemic responses [1,2,3]. Given their ability to minimize the antigen dose and to reduce the number of immunizations needed, emulsion adjuvants (mainly MF59 and AS03) have been the adjuvant of choice during pandemic responses, as seen in the 2009 H1N1 influenza pandemic [4,5,6]. In addition, they have also been extensively investigated and subsequently included in several protein-based vaccines in response to the SARS-CoV-2 pandemic [7,8,9]. Emulsion adjuvants can be manufactured at a large scale in response to increases in demand, but require a complex manufacturing facility, in addition to challenging equipment and maintenance requirements [10]. However, the total availability of these adjuvants may not be sufficient for a full global supply, particularly for lower income countries [4,11]. Hence, a more efficient and cost-effective manufacturing approach is appealing. Although emulsion manufacturing has been tech-transferred to newly established facilities [12,13], simpler methods of manufacturing for these adjuvants may allow pandemic needs to be met for all countries [14,15]. It is established that α-tocopherol acts as an immune potentiator in AS03 and induces better immune responses compared to squalene-only emulsions [16,17]. This was further highlighted by recent work done in the Pulendran lab to evaluate emulsions as adjuvants for a COVID-19 vaccine [18,19]. We have previously shown that a self-emulsification process can be used to make emulsion adjuvants with compositions similar to MF59 [20] and AS03 [14]. Although the α-tocopherol content was low compared to AS03, the self-emulsified adjuvant system 22 (SE-AS22) and SE-AS36 induced humoral and cell-mediated immune responses similar to AS03. However, although influenza antigens are typically the first choice for evaluating novel emulsion adjuvants [6,21,22], the QIV influenza vaccine is a potent immunogen without an adjuvant [23], so does not allow for optimal appraisal of novel adjuvants.

In this paper, we have optimized the SE-AS approach to allow for sterile filtration of the emulsion with minimal changes to critical quality attributes (CQAs) in order to establish the feasibility of its manufacture on a large scale [24]. We also compared AS03 and a squalene-only emulsion (SEA160) [20] with SE-AS44 in human PBMCs to evaluate their ability to activate antigen-presenting cells. Finally, we compared the potency of the optimized SE-AS with AS03 as well as SEA160 to confirm the role of α-tocopherol as an immune potentiator, using the poorly immunogenic Cytomegalovirus (CMV) pentamer recombinant proteinas an antigen. We evaluated the CMV pentamer neutralizing-antibody responses, anti-CMV IgG antibody responses as well as CD4+ T cells and memory T-cell responses in all vaccine groups.

## 2. Materials and Methods

### 2.1. Formulation Materials

Squalene oil was procured from JX Nippon Oil Trading Company (Tokyo, Japan) and dl-α-tocopherol was provided by DSM Nutritional Products (Heerlen, The Netherlands). Polysorbate 80 (PS80) was bought from JT Baker (Center Valley, PA, USA). Hypure (WFI quality) water for injection and phosphate buffered saline (PBS) were procured from HyClone Laboratories (Logan, UT, USA). Tocopheryl polyethylene glycol sulphate (TPGS) was obtained from Antares Healthcare Products, Inc. (St. Charles, IL, USA). CMV pentamer protein was provided by GSK Vaccines (Rockville, MD, USA).

### 2.2. Sterile Filtration of Emulsions

Formulation of SE-AS was carried out as previously described [14]. Emulsions were filtered using a 33 mm, 0.22 µm pore size polyethersulfone (PES) membrane syringe filter from Millipore (Burlington, MA, USA). The resultant filtered emulsions were diluted 500× to measure size and polydispersity index (PdI) using dynamic light scattering (DLS). The % content of squalene and α-tocopherol was evaluated using the method described below.

### 2.3. Percent Content of Squalene and α-Tocopherol in Emulsions

For percent content measurements, reverse phase ultra-high-performance liquid chromatography (RP-UPLC) was used. An Xterra C18 column from Waters (Milford, MA, USA) was used to quantify the oils. Samples were prepared by disrupting emulsions using isopropyl alcohol (IPA) such that the squalene concentration in each emulsion was 200 µg/mL. An amount of 10 µL in duplicate was injected in the UPLC. Mobile phase was 95:5 Methanol:Acetonitrile. The run time was 15 min at 1 mL/min flow rate. The column was heated at 37 °C during elution and a photo-diode array detection (PDA) detector was used to record the eluting peaks. The retention time for tocopherol was ~4.3 min while for squalene it was ~7.4 min. A standard curve of the squalene and tocopherol mixture was run before each run with concentrations ranging from 600 µg/mL to 2.34 µg/mL. Using the slope and intercept from this standard curve, the concentration of squalene and tocopherol in emulsion samples was determined.

### 2.4. Optimization of the SE-AS

Keeping the α-tocopherol content constant at 15% *v*/*v* in oil: surfactant mixture, the polysorbate-80 content in SE-AS emulsions was increased to obtain an SE-AS with a narrower PdI. The goal of this experiment was to obtain an SE-AS which maintains size and PdI post-filtration. The emulsions were formulated similarly as previously described. Measurement of the pH was performed using an Orion 3-star pH meter from Thermo Scientific (Waltham, MA, USA) and osmolality was measured on an Advanced Instruments model 2020 osmometer (Norwood, MA, USA). Size, PdI and percent content of the oils were evaluated using previously described methods.

### 2.5. Cryo-Electron Microscopy (Cryo-EM) Imaging

To understand the morphology of the oil droplets in SE-AS44 compared to AS03, we collaborated with Creative Biostructure (Shirley, NY, USA) to obtain cryo-EM images and size analysis for SE-AS44 and AS03. In summary, the FEI Talos F200C Cryo-Transmission Electron Microscope was used for imaging and analysis purposes. Undiluted emulsion was placed on a thin copper grid that had been glow-discharged and the sample was then loaded into the freezing chamber. The sample was rapidly frozen by plunging into a cryogen (liquid ethane cooled by liquid nitrogen). This frozen sample stored in liquid nitrogen was then used for imaging and size distribution analysis.

### 2.6. In Vitro Innate Immune Response Using Human Whole Blood

To compare the innate immunity activation profile of the SE-AS44 and SEA160 adjuvants with AS03 as standard, an in vitro assay setup was developed where venous whole blood was collected from three healthy volunteers into sodium heparin tubes (Becton Dickinson, San Jose, CA, USA) in accordance with relevant guidelines and regulations, including obtaining an informed consent (ICF Pro00023228, Protocol GSK UM UP BDU). Whole blood was mixed at a 1:1 ratio with RPMI 1640 media and stimulated for 18 h with 1:100, 1:1000, and 1:3000 dilutions of AS03, SE-AS44, and SEA160, respectively. TLR agonists R848 (1 µg/mL), LPS (2 µg/mL), and Poly I:C (20 µg/mL) were used as positive controls, and media and ex vivo stained samples were used as negative controls. The stimulated whole blood samples were stained with fluorescently labeled antibodies to identify leukocytes (anti-CD45-BB515), NKT cells (anti CD3-BV510, anti-CD8-BV786, and anti-CD56-BV650), NK cells (anti CD3-BV510 negative, anti-CD16-BUV496, and anti-CD56-BV650), monocytes (anti CD14-BUV737), mDC and pDC (anti-CD11c-BV421 and anti-CD123-PECy5), and neutrophils (anti-CD66ace-APC), and signals were aac quired on BD Symphony A5 instrument (BD Biosciences, San Jose, CA, USA). The activation status of the cell populations was characterized by measuring the change in the mean fluorescence intensity of scavenger receptor markers (anti CD68-PECy7), T-cell activation co-receptor markers (anti-CD40-BV650, anti-CD86 BB700, and anti-HLA-DR-PECF594), and Fcγ receptor markers (anti-CD16-BUV496, anti-CD32-BUV395, and anti-CD64-BV605). The gating strategy is shown in Supplementary Figure S1a. All antibodies were sourced from BD Biosciences, San Jose, CA, USA except for anti-CD66ace-APC (BioLegend, San Diego, CA, USA), anti CD123-PECy5 (BioLegend, San Diego, CA, USA), anti CD68-PECy7 (eBiosciences, Waltham, MA, USA), and anti CD159a-PE (Beckman Coulter, Brea, CA, USA).

### 2.7. In Vivo Evaluations Using CMV Pentamer Antigen

**Ethics statement:** All animal studies were conducted in accordance with the GSK Policy on the Care, Welfare and Treatment of Laboratory Animals. The protocols were approved by the Institutional Animal Care and Use Committee (IACUC) where the work was performed (Smithers Avanza, Gaithersburg; Approval # D012476). All studies were executed in compliance with provisions of the USDA Animal Welfare Act, the Public Health Service Policy on Humane Care and Use of Laboratory Animals and the U.S. Interagency Research Animal Committee Principles for the Utilization and Care of Research Animals.

**In vivo study design and immunization regimen:** Cell-line-derived CMV pentamer was used to evaluate the in vivo potency of the SE-AS in mice. The dose of antigen in each mouse was 0.05 µg. The antigen content was determined by UV/visible spectrophotometry as well as reverse-phase HPLC. The sample size of 10 animals per group was calculated such that it provided a power of 80% to detect a 3-fold difference between any 2 groups with a 95% confidence interval. Female C57BL/6 mice (aged 6–8 weeks old) from Charles River laboratories (Gaithersburg, MD, USA) were used. The study design comprised of three immunizations three weeks apart, where 50 µL of the vaccine was injected intramuscularly in the gastrocnemius muscle such that, at each subsequent time-point, the alternate limb was used. The CMV pentamer dose in each mouse for each immunization was 0.05 µg. The groups in this study received either: physiological saline (as negative control), unadjuvanted CMV, CMV with SE-AS44, CMV with AS03 or CMV with SEA160.

The vaccine was formulated by mixing the antigen and adjuvant in a 1:1 proportion such that the final antigen concentration reached essentially mimicked that of bedside immunizations. Osmolality, pH, size, PdI and endotoxins were characterized for each formulation before immunization. Endotoxin levels were measured using the Endosafe NexGen–PTS from Charles River laboratories (Wilmington, MA, USA) and limulus amoebocyte lysate (LAL) cartridges with a test range of 0.1–10 EU/mL. Additionally, gel electrophoresis (SDS-PAGE) was used to confirm protein integrity. It was ensured that endotoxin levels for the vaccine used to dose the mice were lower than 1 EU per dose. Bleeds were collected 3 weeks post-1st (3wp1), 3 weeks post-2nd (3wp2) and 3 weeks post-3rd (3wp3) immunization. The processed sera was used to test the antibody responses by neutralizing antibody assay (nAb), a and IgG titers by ELISA. Spleens from 5 animals per group were harvested at the 3wp3 and 4wp3 time-points and used to measure T-cell immune responses via intracellular cytokine staining.

**Determination of neutralizing antibody (nAb) titers:** The retinal pigment epithelial cell line (ARPE-19) was used since TB40 (a CMV virus strain) was known to infect these cells in this assay. On day 1, 100 µL of ARPE-19 cells were plated in 96-well flat-bottom plates in complete growth medium i.e., DMEM + 10% fetal bovine serum (FBS) + 1% Penicillin–Streptomycin. Plates were incubated at 37 °C overnight for ~24 h. On day 2, a Tecan–liquid handling robot was used to perform serum dilutions. Different starting dilutions were used for different time-points depending on the expected titers. A positive control from Sera care known to neutralize the TB40 virus was used in every plate at a constant 1:50 dilution. In each plate, 75 µL of serum dilutions were prepared using Tecan and then 75 µL of TB40 virus was added to each well to make a total of 150 µL in each plate. This was enough for duplicates of each sample. This virus–serum mixture was incubated at 37 °C, 5% CO_2_ for 2 h. The cell plates (duplicates for each group) were removed from the incubator. Media was taken out from each well and 50 µL of virus–serum cocktail was added. These plates were incubated at 37 °C, 5% CO_2_ for at least 20 h. On day 3, the cells were fixed using 4% paraformaldehyde and incubated at RT for 20 min followed by 1 wash using 1xPBS and then permeabilized using 0.1% TritonX-100 and incubated for another 10 min. Primary antibody (anti-mouse anti-CMV IE monoclonal antibody) was added immediately and incubated for 1 h in 37 °C, 5% CO_2_ incubator. Cells were washed twice and then the secondary antibody (anti-mouse AlexaFlour488 antibody) was added and incubated for another 1 h. Post incubation cells were washed 3 times and 1xPBS was added. These plates were then read using high content imaging–CX7 (by selecting to read 10–20 fields per well). Interpolated titers are then calculated at 50% fluorescence intensity.

**IgG antibody ELISA:** Antibody titers were determined in serum obtained from each animal at 3wp2 and 3wp3. To determine the CMV pentamer-specific binding IgG-antibody titers, sandwich ELISA was used. 96-well Nunc-immuno Maxisorp F96 plates were used to coat 100 µL of 1 µg/mL CMV pentamer antigen per well overnight at 4 °C. Antigen-coated plates were washed with 1x phosphate buffered saline (PBS) with 0.05% *w*/*v* Tween20 and blocked with 1% *w*/*v* bovine serum albumin (BSA) solution in PBS. Serum from immunized animals was added to the first row of the plate such that well A1 received serum from the previous CMV study that showed consistently higher titers (positive control) and well A12 received sample buffer as a negative control. The serum was prediluted before adding 10 µL to row1. Serial dilution was then performed down the plate from row A to H. Serum incubation was allowed for one hour before washing the plates and adding horse radish peroxidase (HRP)-conjugated goat anti-mouse IgG from Jackson Immunoresearch (West Grove, PA, USA) for another one-hour incubation at room temperature. Substrate was added quickly after washing plates again, incubated for 15 min and then stop solution was immediately added. Plates were read using EnVision 2105 Multimode plate reader from Perkin Elmer (Waltham, MA, USA). Titers were calculated at 50% interpolated optical density (OD) value obtained from the plate reader.

**Intracellular Cytokine Staining Assay:** Splenocytes from the 4wp3 time point were used to analyze T-cell responses by intracellular cytokine staining. Spleens from individual animals were processed to single-cell suspensions, followed by treatment with RBC lysis buffer (Ebioscience, Thermo Fisher, Waltham, MA, USA). The CMV pentamer peptides gH, gL, UL128, UL130 and UL131 from GeneScript were used for stimulation of splenocytes. These splenocytes were stimulated at a density of one million cells per well using anti-CD3 from BD Biosciences (San Jose, CA, USA) as positive control and media as the negative control, and the peptide pool was prepared for each antigen stimulation condition. Anti-CD28 antibody from BD Biosciences was added to each well as a co-stimulant and brefeldin A (BFA) from BD Biosciences was added two hours after stimulation at 1 µg/mL concentration for blocking cytokine secretion. The cells were stimulated overnight and stained with live/dead reagent (Near IR, EX 633/EM 750). Before the cells were fixed and permeabilized using Cytofix/Cytoperm reagent, Fc block was added to avoid extracellular non-specific binding, followed by memory marker staining using CD62L conjugated with BV510 and CD127 conjugated with BV421 from BD Biosciences. Fc block was again added to avoid intracellular non-specific binding before single-step staining with CD3 conjugated with BV711, IL-17F conjugated with AF647 from BioLegend (San Diego, CA, USA), CD4 conjugated with BUV395, CD8 conjugated with BB700, CD44 conjugated with PEFC594, Interleukin 2 (IL-2) conjugated with APCR700, Interferon γ (IFN-γ) conjugated with BV786, tissue necrotic factor α (TNF-α) conjugated with BV650, IL-17A conjugated with BV421 from BD Biosciences, and IL-13 and IL-4 conjugated with AF488 obtained from Thermo fisher Scientific (Waltham, MA, USA). Since most of the anti-mouse antibodies used are rat or hamster-derived, anti-rat anti-hamster Ig, κ/Negative control compensation particles from BD Biosciences were stained with all the above fluorochrome conjugated antibodies, including an unstained control, for preparing compensation controls. BD FortessaX20 SORP flow cytometer from BD Biosciences (San Jose, CA, USA) was used to acquire the samples followed by analysis with FlowJo software (Ashland, OR, USA). Gating strategy was defined (Supplementary Figure S2a) in FlowJo where the live cells were first differentiated from dead cells, which were then used to differentiate singlets. CD3+ T cells were identified from singlets and used to gate for CD4 and CD8 T cells. Upregulated CD44 cells were used to identify the antigen-specific cells. Individual cytokine gates were then established on these antigen specific CD4 and CD8 T cells. Memory markers were used to identify antigen-specific transitional, central memory, effector memory, and effector populations. Individual cytokine gates were established on these memory populations (Supplementary Figure S2b). Finally, an example of gating for all the media-stimulated, peptide-stimulated and CD3-stimulated cells are shown in Figure S2c.

**Statistics and Data analysis:** GraphPad Prism software (San Diego, CA, USA) was used to analyze, process and graph data from the in vivo immune responses. For humoral responses, one-way analysis of variance (ANOVA) followed by Tukey’s Multiple comparisons test was used to evaluate differences in immune responses from individual animals in the dosing groups; for nAb titers, Dunnett’s test post one-way ANOVA was used. For ICS, a nonparametric Kruskal–Wallis test was run, followed by Dunn’s multiple comparisons test.

## 3. Results

### 3.1. Optimization of the SE-AS Emulsions

Emulsions prepared using a traditional microfluidizer allows a robust production method and enables the emulsion to be sterile-filtered [25]. Therefore it was necessary to show that the SE-AS emulsions were also robust and allowed sterilization by filtration to avoid the need for alternative approaches [24]. Our previous work provided a proof of concept that an emulsion adjuvant containing α-tocopherol could be prepared using self-emulsification, but with lower α-tocopherol content, which still showed non-inferior immune responses in comparison to AS03 [14]. Both emulsions, SE-AS 22 and SE-AS 36, showed comparable immune responses to AS03 and but had size distributions with PdI in the range of 0.2–0.4 [14]. For sterile filtration of a nanoparticle less than 200 nm, it is essential to have a lower PdI [24,26]. We used a PES membrane syringe filter from Millipore to filter 2 mL of the emulsions. Using a method developed on UPLC to quantify squalene and tocopherol in these emulsions, we noticed that both SE-AS 22 and SE-AS 36 showed significant losses in content of the oils post-filtration (Supplementary Table S1). In addition to the loss in content of the oils in the emulsion, there was also an increase in PdI (Supplementary Figure S3), suggesting that these emulsions were not compatible with sterile filtration. Thus, further optimizations were necessary to demonstrate feasibility for sterile filtration.

We have previously reported that the amount and type of surfactant is key to self-emulsification [14,15]. Very high amounts of surfactants showed PdI less than 0.15 but with lesser oil content and lower size (80–100 nm). However, our intent was to optimize the SE-AS while maintaining the size close to AS03 i.e., 155 nm for comparison in vivo. Keeping this in mind, a series of new emulsion combinations (Table 1) were evaluated with varying polysorbate 80 concentrations, keeping tocopherol content between 10–18%. SE-AS14 was used as a starting point owing to its low PdI and tocopherol content in the desired range of evaluation. The trend in general showed that as the surfactant content decreased, the size increased. The increase in PdI was seen in emulsions with higher than 15% *v*/*v* α-tocopherol.

Both SE-AS 44 and 45 showed sizes closest to AS03 and PdI values less than 0.3, which fitted our criteria. These emulsions were then filtered as described previously to evaluate the size post-filtration. Interestingly, SE-AS 45 showed a bimodal size distribution (graph not shown) and increased PdI post-filtration (Table 2). However, SE-AS44 maintained size and showed similar PdI post-filtration (Table 2, Supplementary Figure S4). Further optimization of SE-AS 44 was performed using excipients such as TPGS and poloxamer 188, but the size and PdI did not further improve. In summary, SE-AS 44 was the most optimized emulsion with %*v*/*v* ratio of 60:15:25 Squalene:α-tocopherol:Polysorbate80, as it showed comparable size pre- and post-filtration and maintained the content of the oils within the range of 100 ± 20%. This emulsion was further evaluated for stability at different temperatures for 2 weeks.

**Figure 1:**
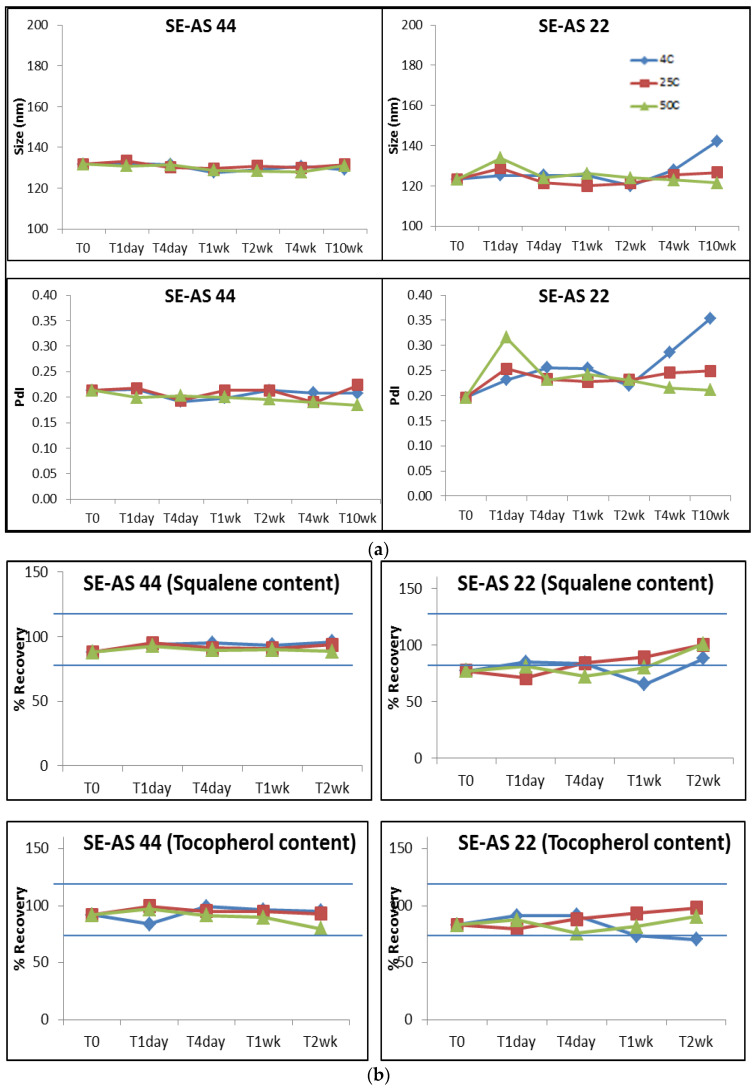

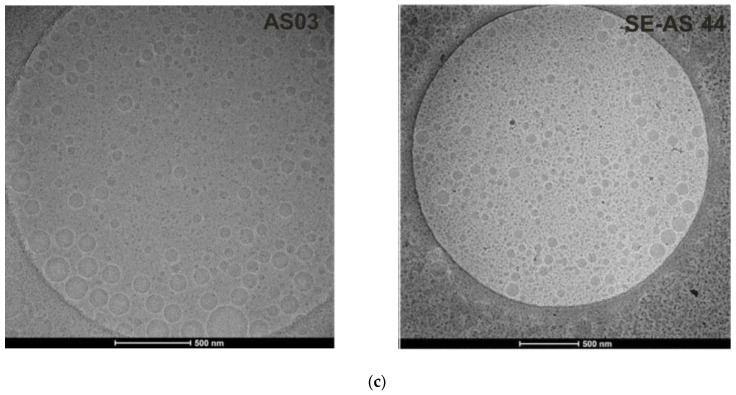
(**a**) Size and PdI for emulsions up to 10 weeks stored at 4 °C (blue), 25 °C (red) and 50 °C (green). (**b**) % Squalene and Tocopherol content for emulsions stored at 4 °C (blue), 25 °C (red) and 50 °C (green) for up to 2 weeks. The blue lines represent the acceptable limits of % content, i.e., 100% ± 20%. (**c**) Cryo-EM images of AS03 and SE-AS44 showing comparable droplet morphology. The oil droplets in AS03 are slightly bigger than SE-AS44, as expected.

Since SE-AS22 had been evaluated previously [14], and is similar in composition to SE-AS44, this emulsion was also subjected to the same stability conditions. Both the emulsions were stored at three different temperatures i.e., 5 °C, 25 °C and 50 °C. Values of pH, osmolality, size and PDI were measured for time-points up to 10 weeks. Percent oil content was also measured at different time-points up to 2 weeks. Osmolality remained in the range for both emulsions (Supplementary Figure S5); however, pH for emulsions stored at 50 °C seemed to drop ~0.3 units. SE-AS 44 showed no change in size and PdI at all temperatures (Figure 1a), unlike SE-AS 22, which showed an increase in size and PdI at 10 weeks. Similarly, SE-AS44 showed both squalene and α-tocopherol content within 80–120% which was comparable at all time points up to 2 weeks (Figure 1b), making it the selected emulsion candidate for further evaluation. Before in vitro and in vivo evaluations, SE-AS44 was compared to AS03 using Cryo-EM for droplet morphology and size. The droplet morphology of SE-AS44 was similar at a 500 nm scale to AS03. The size distribution and analysis for SE-AS44 showed that the majority of the droplets (~87% considered in the analysis) were less than 150 nm in size (Figure 1c).

### 3.2. In Vitro Evaluation of Novel Emulsion Adjuvants Using Human Innate Signaling in Whole Blood Cells

**Figure 2:**
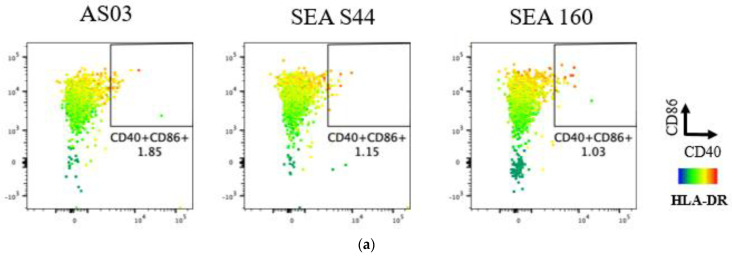

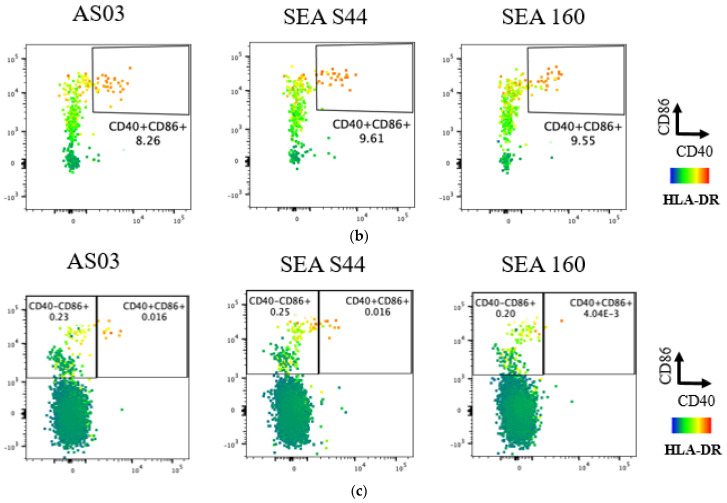
Representative pseudo-color plots showing the tri-variate analysis of CD40 vs. CD86 markers with characterization of HLA-DR expression using heatmap analysis of human whole blood samples stimulated with adjuvants (AS03, SEA S44, and SEA160) with media-treated and ex vivo stained samples as negative controls. AS03, SEA S44, and SEA160-stimulated whole blood samples showed an increase in CD40, CD86, and HLA-DR markers compared to media-treated samples in monocytes (**a**), mDC (**b**), and neutrophils (**c**), indicating induction of an antigen-presentation role.

Prior to evaluating SE-AS44 in vivo, the role of α-tocopherol was evaluated by comparing in vitro readouts with SEA160, an optimized squalene-only emulsion [20]. Among different immune cell populations, T cell co-receptor markers CD40, CD86, and HLA-DR were upregulated primarily in monocytes, mDC and neutrophils (Figure 2) in whole blood samples stimulated with media, AS03, SEA S44, and SEA160. These receptor markers have been previously shown to induce antigen presentation capability [27]. SEA160 showed similar levels of CD40+CD86+ monocytes and mDCs compared to AS03 and SE-AS44. However, overall levels of CD40+CD86+ neutrophils were low for all adjuvants; SE-AS44 and AS03 showed four times higher levels compared to SEA160. Similar observations between AS03 with and without tocopherol have been reported previously [16]. Following this qualitative analysis in the human whole blood cells in vitro, the emulsions were evaluated in mice using CMV pentamer as the model antigen.

### 3.3. In Vivo Potency of Emulsion Adjuvants in Mice Using CMV Pentamer Antigen

The goal of the in vivo study was to compare the optimized SE-AS with the well-characterized squalene-only emulsion SEA160, using a soluble subunit protein with inherently low immunogenicity. AS03 was used as a control. The groups are as described in the materials and methods section.

**Figure 3:**
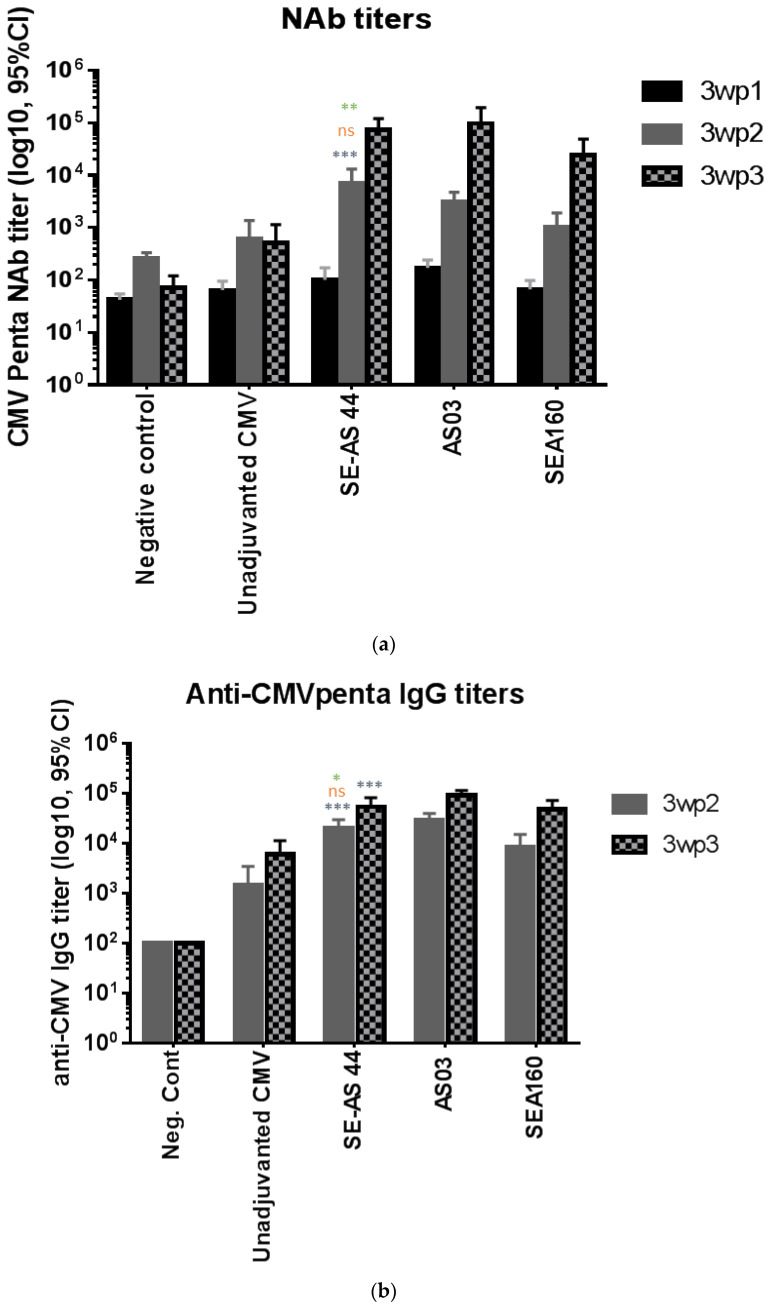
(**a**) Neutralizing antibody titers in serum obtained three weeks post-1st, 2nd and 3rd immunization against the CMV TB40 strain of virus. Each bar represents geometric mean titers (GMT) with 95% confidence interval (CI) from *n* = 10 animals per group. Statistical analysis was performed using one-way ANOVA followed by Tukey’s test to compare all groups with each other followed by Dunnett’s multiple comparisons test to compare each group with SE-AS44. Significant differences are marked on the graph only for 3wp2 titers. Comparison with CMV alone is shown in blue, with AS03 is shown in red, and with SEA160 is shown in green; ns = not significant, ** = *p* ˂ 0.005, *** = *p* ˂ 0.0005 . (**b**) Anti-CMV Penta IgG antibody titers in serum obtained three weeks post-1st, 2nd and 3rd immunization against the CMV TB40 strain of virus. Each bar represents geometric mean titers (GMT) with 95% confidence interval (CI) from *n* = 10 animals per group. Statistical analysis was performed using one-way ANOVA followed by Dunnett’s multiple comparisons test to compare differences between each group with SE-AS44. Significant differences are marked on the graph. Comparison with CMV alone is shown in blue, with AS03 is shown in red and with SEA160 is shown in green; ns = not significant, * = *p* ˂ 0.05, *** = *p* ˂ 0.0005.

Although the primary readout was neutralizing antibodies (nAb), anti-CMV IgG antibodies as well as specific CD4+ T-cell activation were also evaluated. Titres of nAb (Figure 3a) three weeks after the 1st immunization (3wp1) were low owing to the inherently low immunogenicity of the antigen, even though adjuvanted groups showed slightly higher titers. At 3wp2, there was a significant increase in titers for adjuvanted groups compared to unadjuvanted or negative controls. SE-AS44 showed significantly higher titers compared to unadjuvanted protein, but not significantly different to AS03. Most importantly, a significant difference was observed between SE-AS44 and SEA160 titers at 3wp2. At 3wp3, the trend remained similar, with little to no increase in titers for unadjuvanted protein, but SE-AS44 showed significantly higher titers compared to the unadjuvanted group. Mean titers for SE-AS44 were higher compared to SEA160 but were not very different compared to AS03.

Binding antibody titers in sera obtained from 3wp2 and 3wp3 time-points were measured by IgG ELISA. Mean titers showed an overall trend between the groups similar to that for the nAb titers (Figure 3b). All adjuvants showed significantly higher titers compared to CMV protein alone. AS03 gave the highest titers post-3rd immunization. However, significant differences were observed for SE-AS44 compared to SEA160. Thus, the overall humoral response favored adjuvanted groups, with SE-AS44 showing evidence of higher titers post-2nd immunization and overall higher mean IgG and nAb titers compared to SEA160. This data suggests that α-tocopherol may be playing a key role in improving the humoral immune response compared to squalene-only emulsion adjuvants.

**Figure 4:**
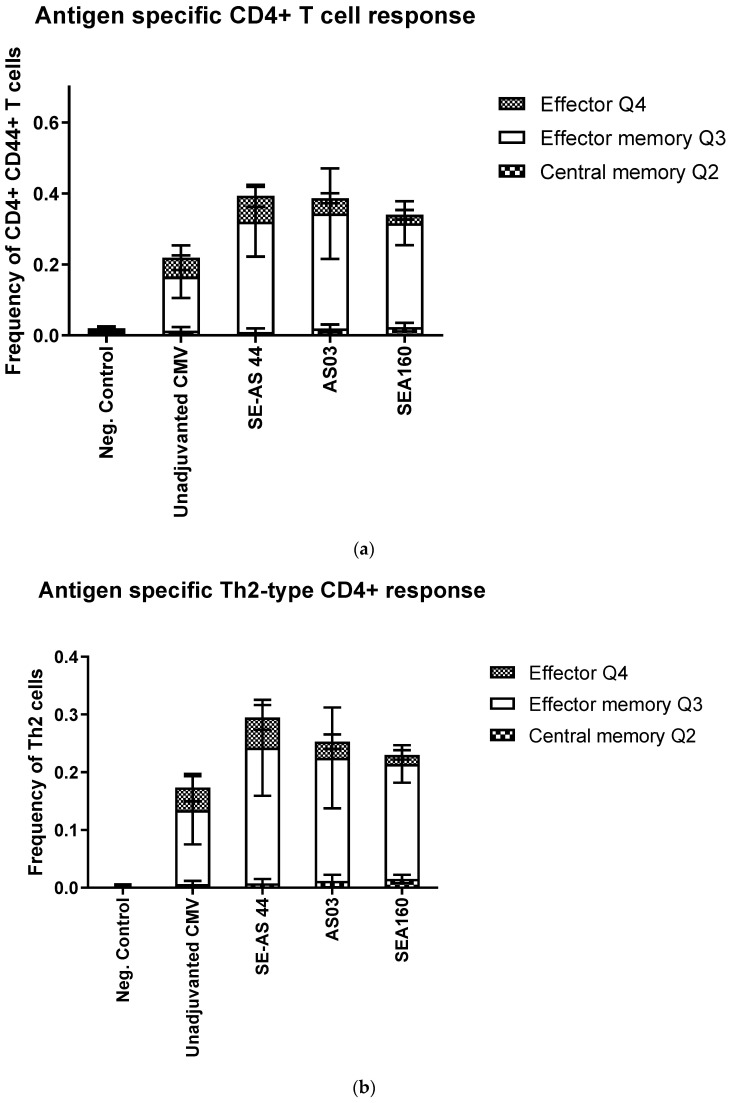

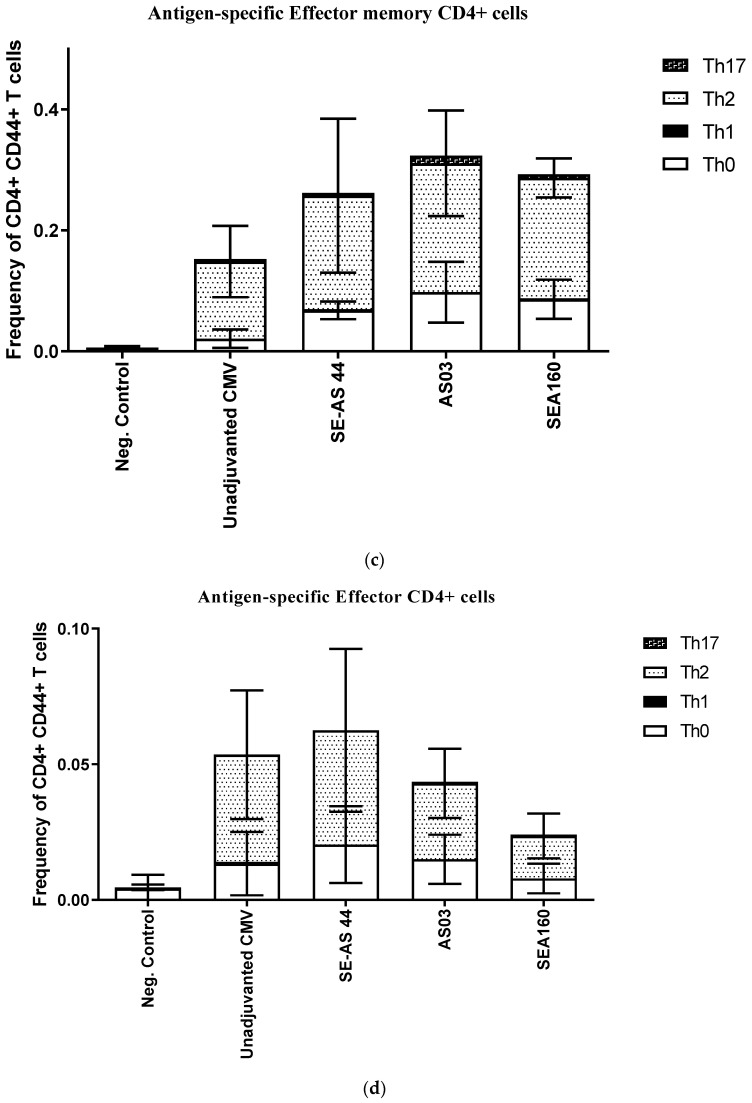
(**a**) Frequencies of overall antigen-specific CD4+ T cells classified as either central memory, effector memory or effector cells. Each bar represents stacked mean frequency data from *n* = 5 animals with standard deviation. Supplementary Table S2a shows frequency of cells for each group. (**b**) Frequencies of antigen-specific Th2-type CD4+ T cells classified as either central memory, effector memory or effector Th2 cells. Each bar represents stacked mean frequency data from *n* = 5 animals with standard deviation. Supplementary Table S2b shows frequency of cells for each group. (**c**) Frequencies of antigen-specific effector memory cells gated from Quadrant 3 after gating for memory markers. Each bar represents stacked mean frequency data from *n* = 5 animals with standard deviation. Supplementary Table S2c shows frequency of cells for each group. (**d**) Frequencies of antigen-specific effector cells gated from Quadrant 4 after gating for memory markers. Each bar represents stacked mean frequency data from *n* = 5 animals with standard deviation. Supplementary Table S2d shows frequency of cells for each group.

Finally, to determine cell-mediated immune responses, spleens from immunized mice 4 weeks post-3rd immunization were evaluated. We used the memory markers CD62L and CD127 to differentiate the CD4+ and CD8+ T cells into transitional, central memory, effector memory and effector cells. Prophylactic vaccines are designed to induce an efficient memory T-cell response against pathogens, which can eventually help the effector response [28,29]. Splenocytes were stained for both CD4+ and CD8+ T cells; however, the frequency of CD8+ T cells was very low, so only CD4+ T cell data was characterized. There were few to no transitional cells, the most frequent T cells were either effector or effector memory cells. Effector memory cells prevailed as the majority of CD4+ T cells when these were gated to look at the memory response (Figure 4a). There were no significant differences between the different adjuvant groups, although SE-AS44 showed higher total frequency of CD4+ T cells. As expected, when the CD4+ cells were gated into subtypes, Th2-subtype prevailed over others. The Th2-type response in mice is characteristic of emulsion adjuvants [10,14,30,31] and SE-AS44 showed the highest frequency of cells (Figure 4c). Although SE-AS44 shows slightly lower effector memory CD4+ T cells, our hypothesis is that this decrease in effector memory cells may have translated into a higher frequency of effector cells (Figure 4d).

## 4. Discussion

Previously we showed, using a Quadrivalent influenza antigen, that SE-AS adjuvanted emulsions induced non-inferior immune responses compared to AS03 in mice. While incorporating α-tocopherol into SE-AS to obtain a stable emulsion was a significant formulation challenge, we established the feasibility of creating an SE-AS with tocopherol; however, the amount of α-tocopherol was reduced to 15%v/v compared to 42% *v*/*v* in AS03. Here, to advance this work, we further optimized the SE-AS formulations to improve the filterability of the adjuvants, and to demonstrate short-term stability at accelerated storage conditions. The optimized candidate, SE-AS44, was stable even at 50 °C for up to two weeks. Additionally, the morphology of these droplets was similar to that of AS03, suggesting that there was no impact of the process of formulation on overall droplet morphology. In vitro experiments with whole blood cells allowed SE-AS44 to show higher levels of neutrophil activation compared to SEA160, a squalene-only emulsion, which was consistent with previous findings [16]. SE-AS44 was selected as the optimized candidate for evaluation of potency with the CMV pentamer, which is a soluble recombinant protein expressed in CHO cells [32]. CMV pentamer alone has very poor immunogenicity [32] and was an ideal candidate to evaluate the role of adjuvant. The in vivo study showed that all adjuvants significantly improved the humoral immune response after the second immunization. The SE-AS44 adjuvanted groups showed significantly higher nAbs as well as anti-CMV IgG antibodies compared to the SEA160 groups. This data confirmed the role of α-tocopherol as an immune potentiator in emulsion adjuvants, which is consistent with previous data [16] and [19]. As expected, the emulsion adjuvants overall showed a Th0/Th2-biased T-cell response. Within the Th2 population, a higher frequency of effector cells was observed for SE-AS44 compared to other emulsions.

## 5. Summary

Emulsion-adjuvanted vaccines have been at the forefront of vaccine developments in response to a pandemic [4,7,10,33]. This work provides a proof-of-concept for potentially developing these emulsion adjuvants using a much simpler process, which may expedite production globally, including for low-income countries. In addition, we showed that α-tocopherol acts as an immune potentiator in squalene-based emulsion adjuvants, and that it can also be incorporated using a self-emulsification process, although at lower concentrations than present in AS03.

## Figures and Tables

**Table 1:** % *v*/*v* in oil: surfactant mixture for novel SE-AS combinations and size (nm) and PdI using DLS. SE-AS 22 is shown for comparison.

SE-AS	Tween80 (%*v*/*v*)	Squalene (%*v*/*v*)	α-Tocopherol (%*v*/*v*)	Avg Size (nm)	PdI
14	35	55	10	79.76	0.113
41	33	55	12	94.13	0.166
42	30	55	15	120.73	0.219
43	30	52	18	134.20	0.360
44	25	60	15	131.73	0.192
45	20	65	15	139.10	0.215
22	15	70	15	129.07	0.212
AS03	16	42	42	155	˂0.2

**Table 2:** Size, PdI and percent content loss pre- and post-filtration for SE-AS44 and 45.

SE-AS	Before Filtration		After Filtration		% Content Loss	
	Size	PdI	Size	PdI	Squalene	α-Tocopherol
44	133.7	0.184	134.9	0.203	16.3	12.64
45	141.5	0.201	177.1667	0.329	-	-

## Data Availability

Data will be made available upon request.

## Supplementary Materials

The following supporting information can be downloaded at: https://www.mdpi.com/article/10.3390/pharmaceutics15010238/s1.
