# Supplementary material for: A Self-Emulsified Adjuvant System Containing the Immune Potentiator Alpha Tocopherol Induces Higher Neutralizing Antibody Responses than a Squalene-Only Emulsion When Evaluated with a Recombinant Cytomegalovirus (CMV) Pentamer Antigen in Mice
Source: Pharmaceutics. 2023 Jan 10;15(1):238. doi: 10.3390/pharmaceutics15010238 (PMC9867524; doi:10.3390/pharmaceutics15010238)
Supplement: Supplementary file 1 [file pharmaceutics-15-00238-s001.zip › pharmaceutics-2036149-supplementary.pdf]

Supplementary Figures and Tables

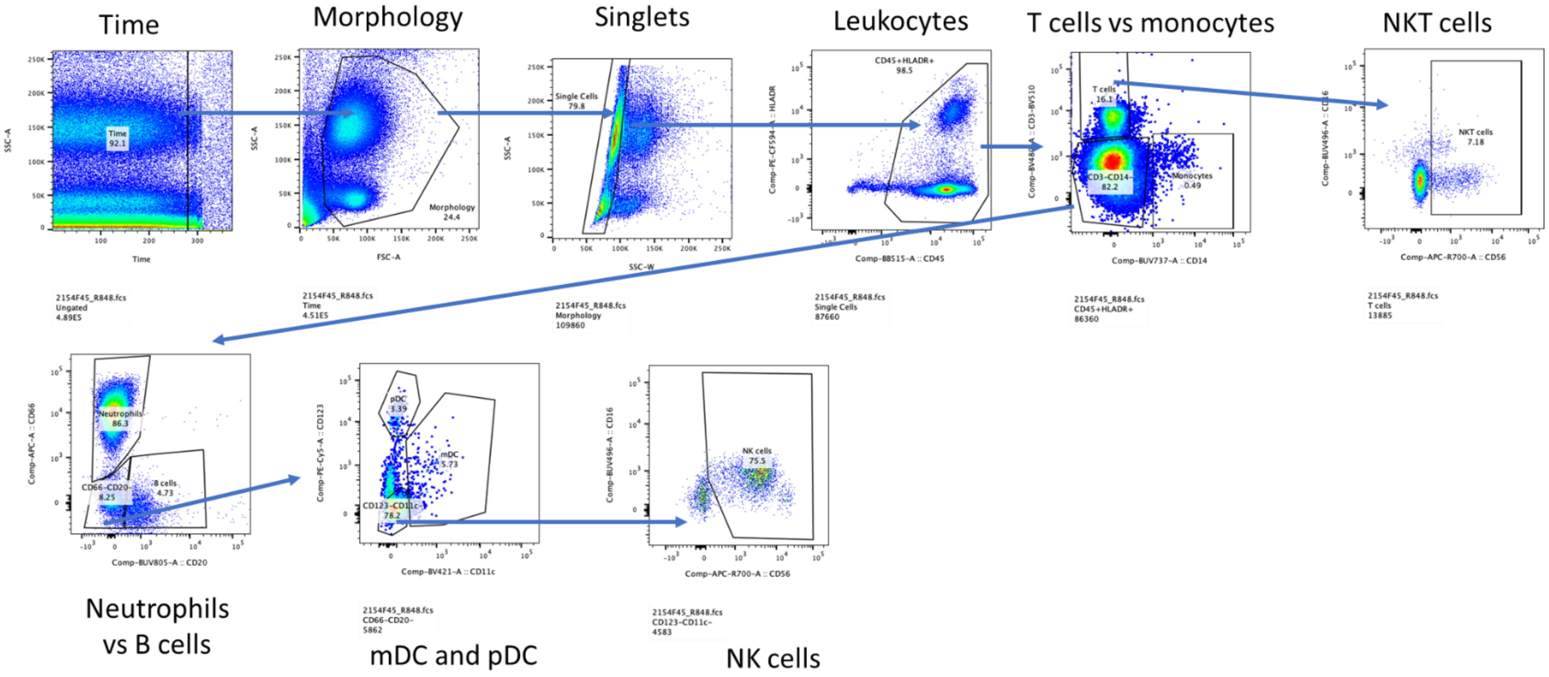

(**a**)

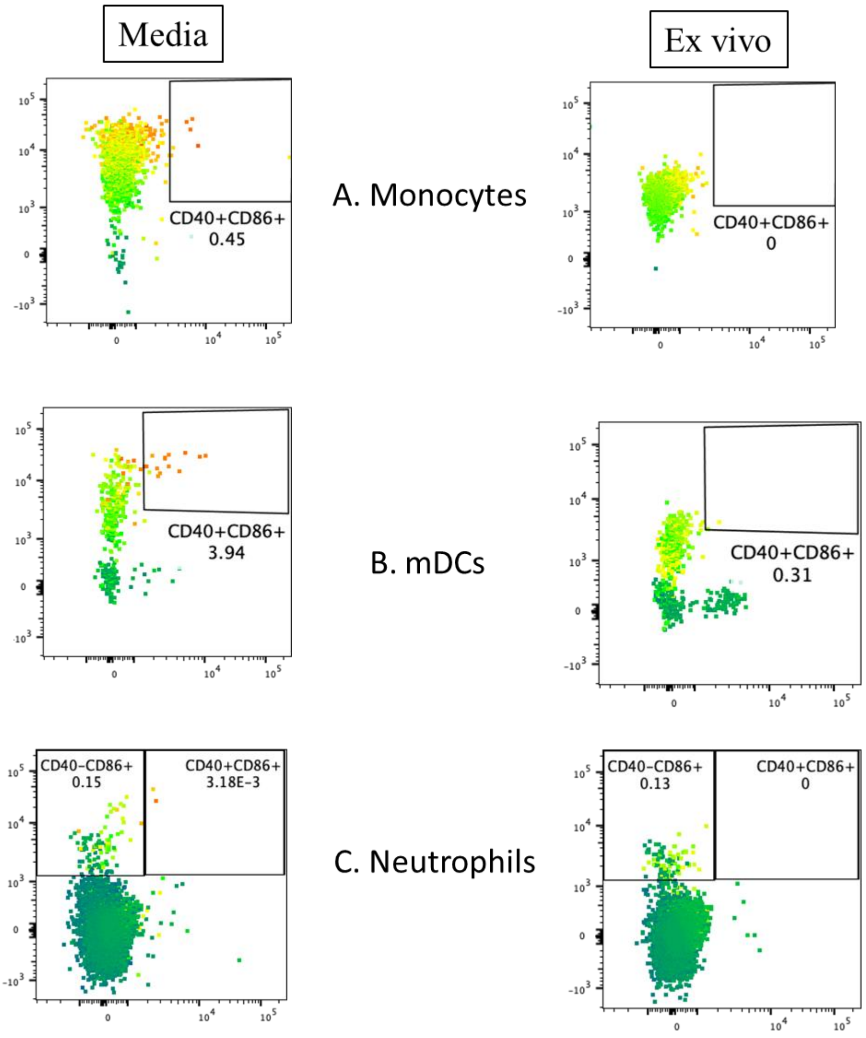

(**b**)

Figure S1. (**a**): Example of gating strategy for in vitro staining of human whole blood cells. T cells vs monocytes are identified after gating the leukocytes. Monocytes are further gated into Neutrophils, mDCs and NK cells. (**b**): Media and ex vivo staining for each cell type from Monocytes (A), mDCs (B) and Neutrophils (C).

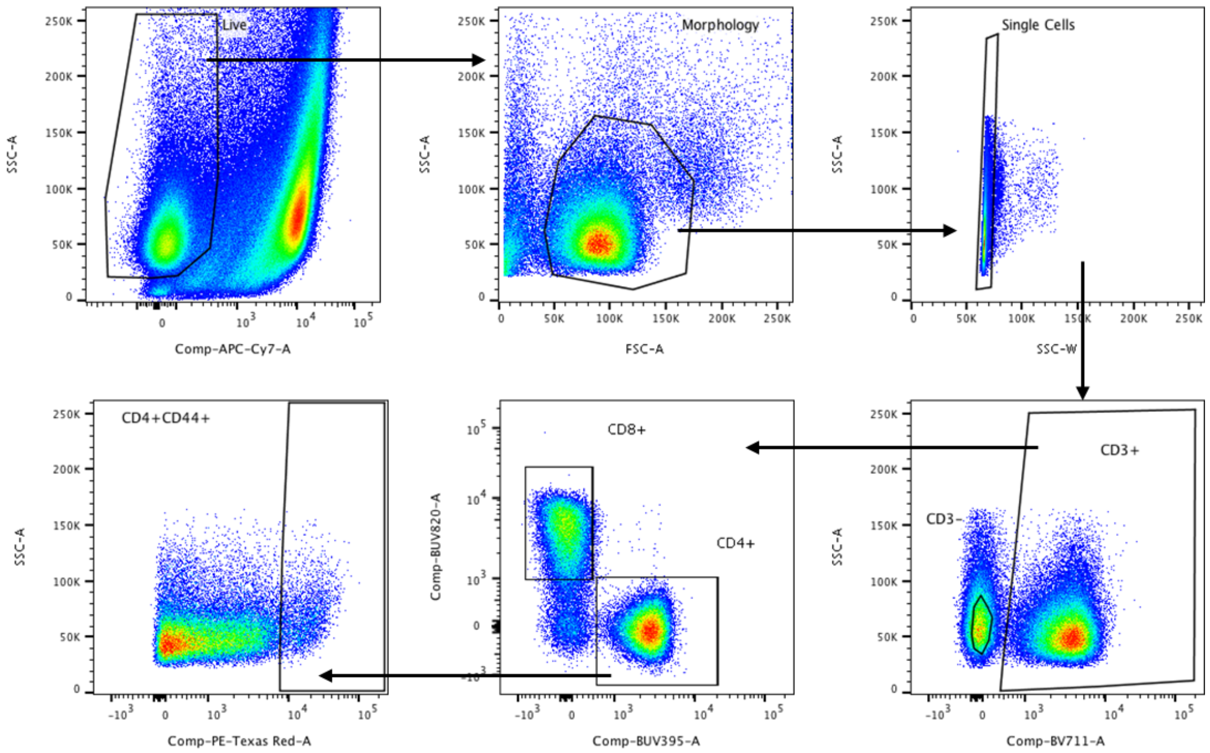
Live
Morphology
Single Cells
SSC-A
Comp-APC-Cy7-A
FSC-A
SSC-W
CD4+CD44+
CD8+
CD4+
CD3+
CD3-
Comp-BUV820-A
Comp-PE-Texas Red-A
Comp-BUV395-A
Comp-BV711-A

(**a**)

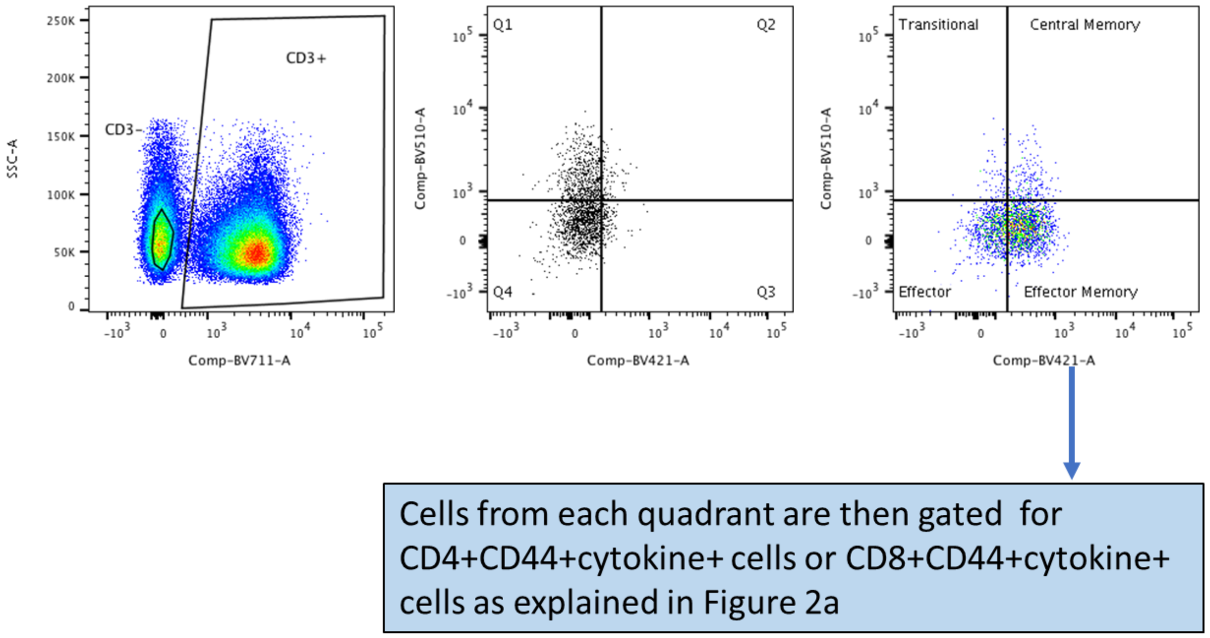
CD3+
CD3-
SSC-A
Comp-BV711-A
Q1
Q2
Q3
Q4
Comp-BV510-A
Comp-BV421-A
Transitional
Central Memory
Effector
Effector Memory
Cells from each quadrant are then gated for CD4+CD44+cytokine+ cells or CD8+CD44+cytokine+ cells as explained in Figure 2a

(**b**)

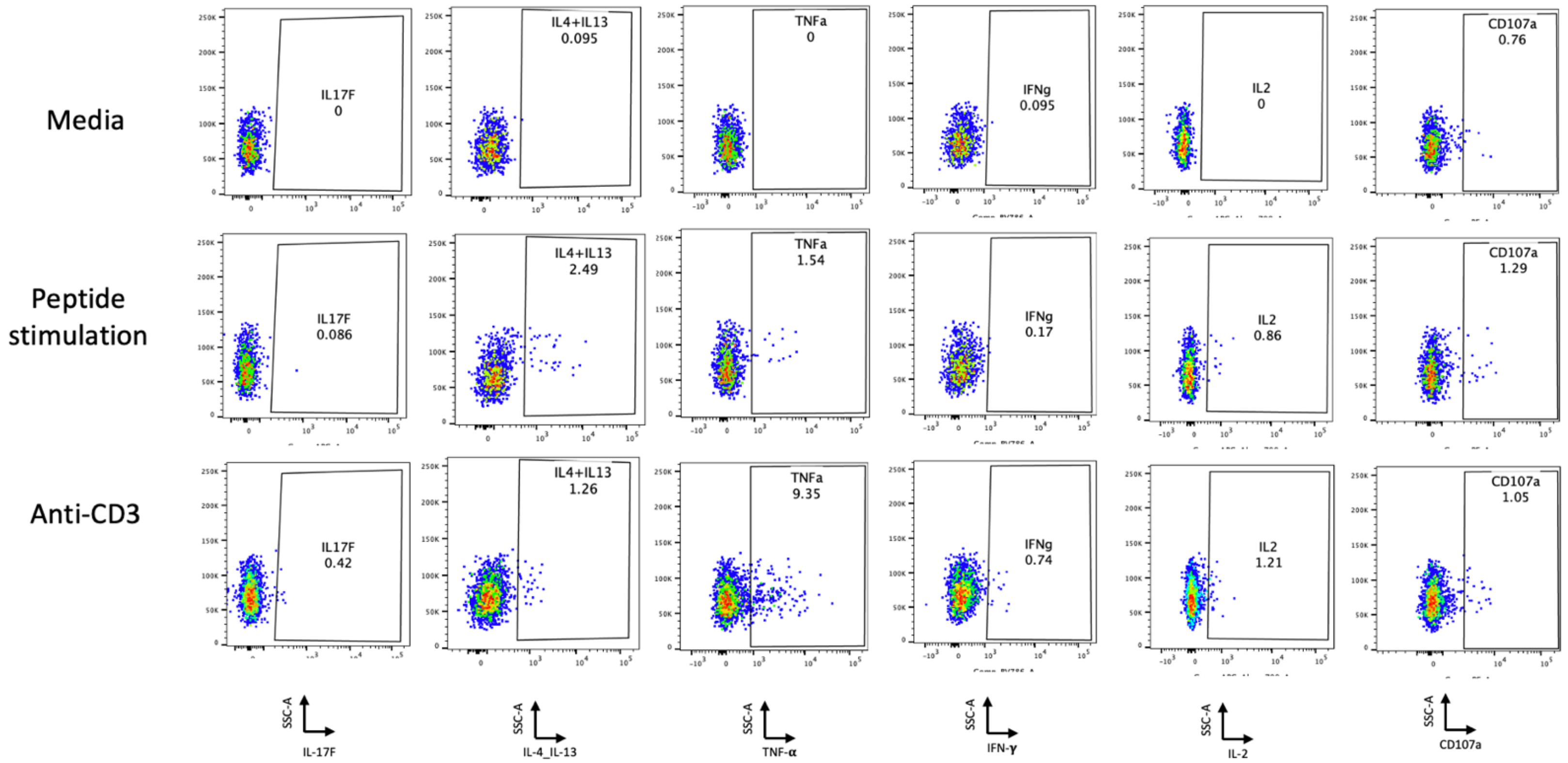

(**c**)

Figure S2. (**a**): Example of gating strategy for ICS. First live cells are differentiated from dead, and then separated based on morphology to get single cell lymphocytes, differentiated based on CD3 marker. CD4+ and CD8+ T cells are then gated based on CD3 gates followed by identifying antigen specific CD4+ or CD8+ T cells. (**b**): Example of gating strategy for Memory markers. CD3- cells are used to gate four quadrants of T lymphocyte cells based on CD62L and CD127 markers. Q1 represents transitional T cells, Q2 represents Central Memory T cells, Q3 represents Effector memory and Q4 represents Effector cells. (**c**): Representative ICS staining figure showing gating for media control, peptide stimulated cells and anti-CD3 control.

SE-AS	Before filtration		After filtration		% Content loss after filtration	
	Size	PdI	Size	PdI	Squalene	Tocopherol
**22**	135.2	0.241	230.1	0.484	31.12	32.38
**36**	173.3	0.283	371.3	0.619	45.12	61.02

Table S1. Size of the emulsions before and after filtration and % content loss after filtration for SE-AS 22 and 36.

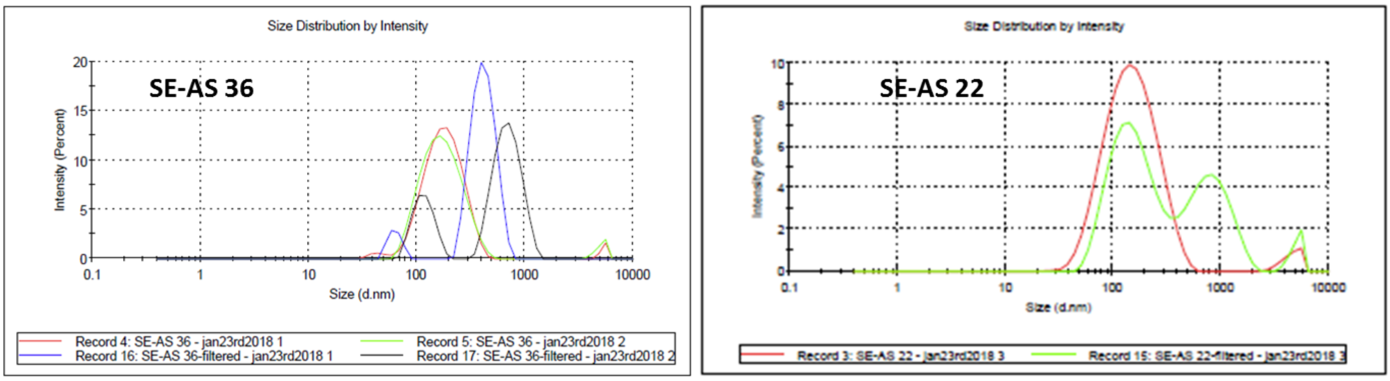

Figure S3. Bimodal size distribution after filtration through 0.22µ PES filter. Graphs in red & green for SE-AS 36 are before filtration, and those in blue and black are post filtration. For SE-AS 22, red is before and green is after filtration.

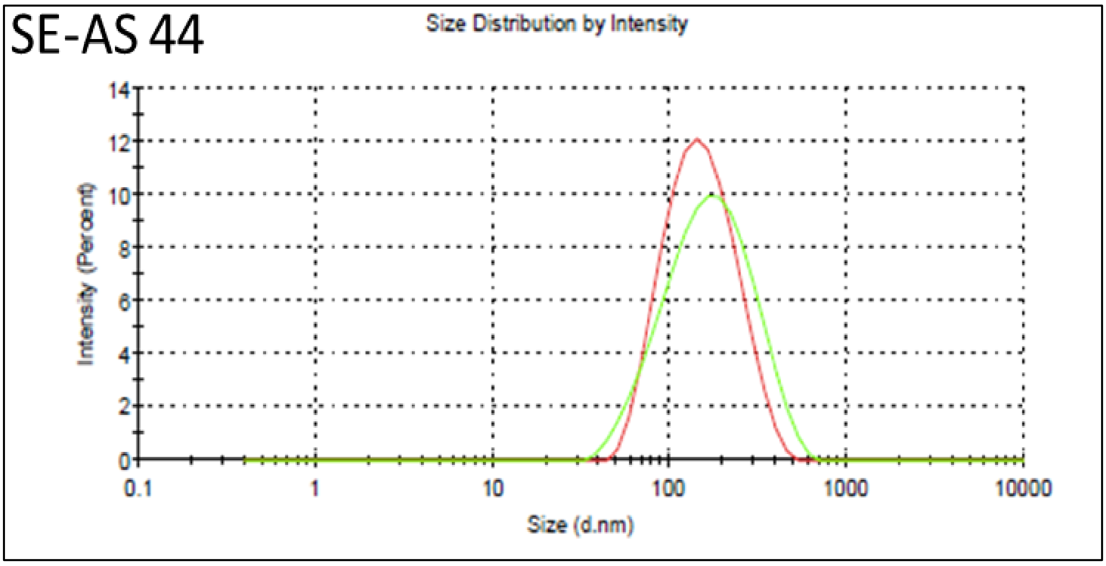

Figure S4. Size distribution by intensity for SE-AS 44, showing distribution pre- (red curve) and post- (green curve) filtration.

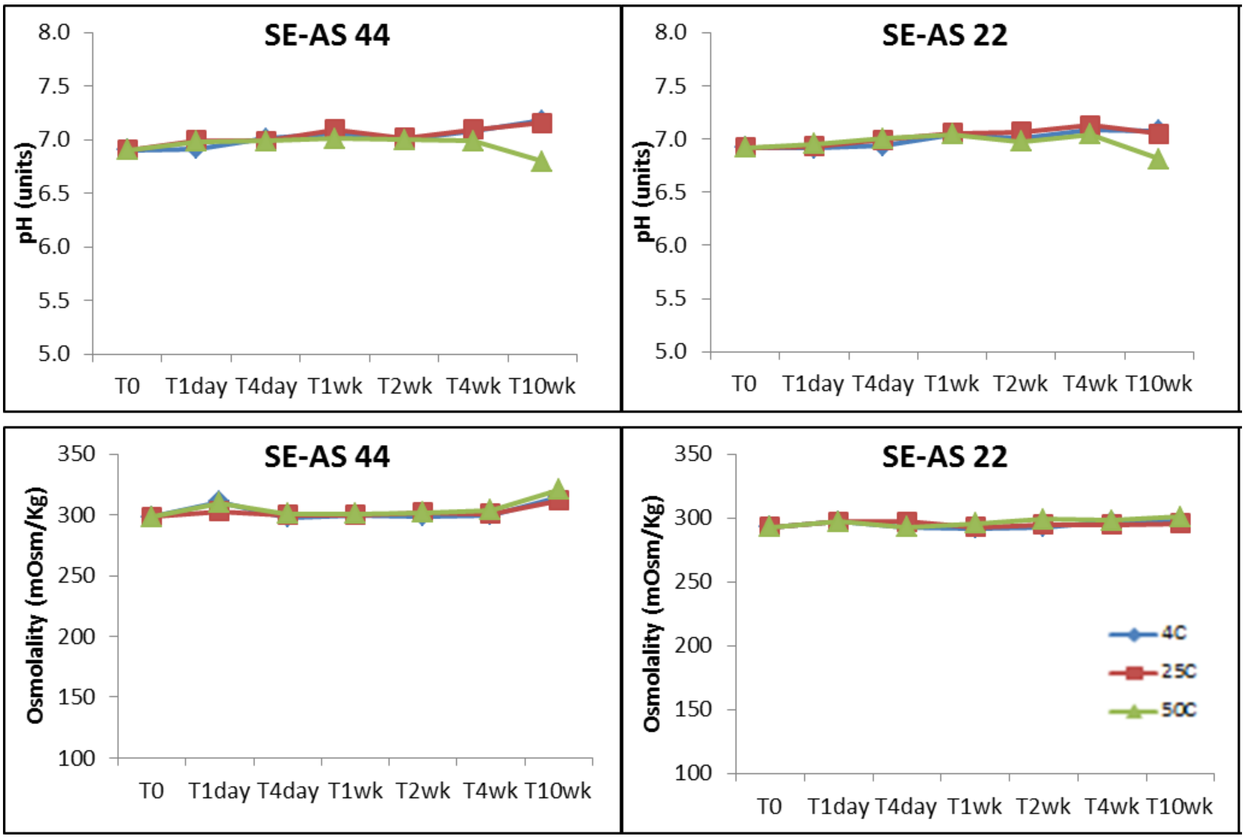

Figure S5. pH and Osmolality for emulsions stored at 4C (blue), 25C (red) and 50C (green) for up to 10 weeks.

(a) Overall CD4+ T cells	CM Q2	EM Q2	E Q4	
**Neg. Control**	0.010	0.006	0.005	
**Unadjuvanted CMV**	0.013	0.152	0.054	
**SE-AS 44**	0.010	0.310	0.073	
**AS03**	0.020	0.323	0.044	
**SEA160**	0.023	0.293	0.024	
(b) **Th2-type CD4+ T cells**	**CM Q2**	**EM Q2**	**E Q4**	
**Neg. Control**	0.002	0.000	0.000	
**Unadjuvanted CMV**	0.006	0.128	0.039	
**SE-AS 44**	0.008	0.235	0.053	
**AS03**	0.012	0.213	0.028	
**SEA160**	0.015	0.199	0.016	
(c) **EFFECTOR MEMORY CD4+ T cells**	**Th0**	**Th1**	**Th2**	**Th17**
**Neg. Control**	0.004	0.000	0.000	0.002
**Unadjuvanted CMV**	0.021	0.000	0.128	0.004
**SE-AS 44**	0.068	0.002	0.188	0.004
**AS03**	0.098	0.000	0.213	0.013
**SEA160**	0.086	0.002	0.199	0.006
(d) **EFFECTOR CD4+ T cells**	**Th0**	**Th1**	**Th2**	**Th17**
**Neg. Control**	0.004	0.000	0.000	0.000
**Unadjuvanted CMV**	0.013	0.001	0.039	0.000
**SE-AS 44**	0.020	0.000	0.042	0.000
**AS03**	0.015	0.000	0.028	0.001
**SEA160**	0.008	0.000	0.016	0.001

Table S2. Frequency of antigen-specific CD4+ T cells corresponding to figure 4 in overall CD4+ T cell population further characterized into memory T cells (**a**), Th2-type CD4+ T cells (**b**), overall effector memory T-cells (**c**) and effector cells (**d**).
